# Screening strategies for the diagnosis of asymptomatic *Leishmania* infection in dialysis patients as a model for kidney transplant candidates

**DOI:** 10.1007/s40620-020-00705-4

**Published:** 2020-01-30

**Authors:** Giorgia Comai, Alessandra Mistral De Pascali, Marco Busutti, Silvia Morini, Margherita Ortalli, Diletta Conte, Maria Carla Re, Gaetano La Manna, Stefania Varani

**Affiliations:** 1 Department of Experimental, Diagnostic and Specialty Medicine (DIMES)–Nephrology, Dialysis and Renal Transplant Unit, St. Orsola-Malpighi Hospital, University of Bologna, Via G. Massarenti 9 (Pad. 15), 40138 Bologna, Italy; 2 Department of Experimental, Diagnostic and Specialty Medicine, University of Bologna, Bologna, Italy; 3 Unit of Clinical Microbiology, Regional Reference Centre for Microbiological Emergencies (CRREM), St. Orsola-Malpighi University Hospital, Bologna, Italy

**Keywords:** *Leishmania infantum*, End stage renal disease, Dialysis treatment, Serological methods, Molecular methods

## Abstract

Despite being considered a tropical disease, visceral leishmaniasis (VL) caused by *L. infantum* is also endemic in the Mediterranean Europe and represents an increasing cause of morbidity and mortality in solid organ transplant (SOT) recipients. VL occurring in kidney transplant recipients is a severe event, often worsening the renal damage and leading to poor outcome. It is believed that most of VL cases in transplant recipients are caused by reactivation of a pre-existent, dormant leishmanial infection induced by the immunosuppressive drugs. Nevertheless, the prevalence of asymptomatic *Leishmania* infection in candidates to kidney transplant residing in or visiting endemic areas is unknown. As *L. infantum* is highly circulating in northeastern Italy, we aimed to examine the occurrence of this parasitic infection in 119 dialysis patients living in the mentioned area, 71 of whom were potential candidates to kidney transplant. By employing a combination of sensitive serological and molecular methods, we observed a prevalence of 15.9% asymptomatic *Leishmania* infection in the study cohort. This finding emphasizes the need of further evaluating potential screening strategies for *Leishmania* infection in solid organ transplant candidates residing in or visiting endemic areas.

## Introduction

Leishmaniasis is a chronic infectious disease caused by protozoa of the genus *Leishmania* [1]. The parasites are spread through the bite of infected phlebotomine sand flies and mostly target reticuloendothelial cells. The clinical spectrum of disease depends on the parasite species and on the host immune response, varying from asymptomatic infection (80–95% of infected individuals) to three different clinical manifestations (5–20% of cases): visceral leishmaniasis (VL), cutaneous leishmaniasis and mucosal or mucocutaneous leishmaniasis. VL is a severe disease caused by *Leishmania* species belonging to the *Leishmania donovani* complex; in the Mediterranean Europe, VL is caused by *L. infantum.* Immunosuppression may reactivate latent leishmanial infection [2] and among HIV-infected patients the risk of clinical VL is increased by over 100 times. In recent years, an increase of VL cases has also been reported in solid organ transplant (SOT) recipients [3, 4].

Among immunocompromised patients, cutaneous and mucocutaneous leishmaniasis are rare, whereas VL is the dominant clinical presentation [4]. The prevalence of VL among SOT recipients is 0.05–0.9% in endemic areas [5] and more than 100 VL cases following transplantation—mainly kidney transplant—have been reported worldwide up to 2017 [6]. The classic triad of fever, splenomegaly, and pancytopenia only occur in one third of SOT patients, leading to difficulties in diagnosis, in particular, splenomegaly is reported less frequently than in immunocompetent patients [4, 6]. In kidney transplant recipients, leishmaniasis often includes acute interstitial nephritis with moderate inflammation and infiltration of lymphocytes, plasma cells and macrophages and can lead to graft dysfunction [7]. Moreover, VL in SOT exhibits high relapse and mortality rates [2, 3]. The most common cause of death in immunocompromised patients with VL is the presence of concomitant bacterial, viral or fungal infections [4, 8].

Currently, screening of the candidates to SOT for *Leishmani*a infection in endemic areas is not recommended [2, 5, 9], but a strict monitoring is suggested in case a *Leishmania*-positive recipient is found for potential reactivation of this parasitic infection [6].

The aim of this study was to investigate the presence of clinically silent *Leishmania* infection in candidates to SOT. We analyzed the prevalence of asymptomatic *Leishmania* infection by serological and molecular tests in a cohort of patients with end stage renal disease (ESRD) undergoing dialysis treatment, most of whom were potential candidates to kidney transplant. The study was performed in the Bologna province, northeastern Italy, which is an endemic area for human leishmaniasis [10].

## Methods

### Study population, setting, and samples

We conducted a retrospective study including a target population of ESRD adult patients in dialysis treatment. Inclusion criteria were: (1) absence of medical history of VL or cutaneous leishmaniasis, (2) lack of symptoms for VL or suspected lesions for cutaneous leishmaniasis at the time of blood sampling, (3) absence of other infectious diseases at the time of blood sampling, and (4) residence in the Bologna province. Among the 119 patients included for the study (92% Caucasians, 61.3% male, 38.7% female, mean age 70 years), 71 patients (60%) were potential candidates to kidney transplant. Serum and whole blood samples were collected from May to December 2016 at the Nephrology, Dialysis and Renal Transplantation Unit, St. Orsola-Malpighi University Hospital (Bologna, Italy) and stored at  − 80 °C until analysis. The samples were subsequently investigated for the presence of anti-*Leishmania* antibodies and leishmanial DNA. The study was conducted in accordance with the Declaration of Helsinki, and the protocol was approved by the Ethics Committee of the St. Orsola-Malpighi University Hospital (project identification code: 97/2017/O/Tess).

### Detection of anti-*Leishmania* antibodies

The presence of specific anti-*Leishmania* IgG was assessed by Western Blot (WB). All sera were analyzed by a commercially available Leishmania Western Blot IgG (LDBio Diagnostics®, Lyon, France) according to the manufacturer’s recommendations. WB technique provides detailed antibody responses to the leishmanial 14-kDa and 16-kDa proteins of *L. infantum*. The presence of specific antibody to p14 and/or p16 proteins was considered as a positive result [11].

### Detection of parasitic DNA

A PCR assay based on kinetoplast (k)DNA amplification was developed for detection of *Leishmania* DNA in whole blood samples. Nucleic acids were extracted from 100 µL whole blood with NucliSENS*easyMAG *(*Biomerieux,* Marcy l’Etoile, France)*.* DNA was eluted in 50 µL of elution buffer. DNA was amplified employing a PCR targeting leishmanial kinetoplast (k)DNA [12]. Primers (15 pmol of RV1 5′-CTTTTCTGGTCCTCCGGGTAGG-3′, 15 pmol of RV2 5′-CCACCCGGCCCTATTTTACACCAA-3′) were synthesized by PrimmBiotech (Milan, Italy) and 50 pmol of TaqMan probe (FAM-TTTTCGCAGAACGCCCCTACCCGC-TAMRA) were synthesized by IDTDNA (Leuven, Belgium). β2-microglobulin Real-time PCR assay was run simultaneously as a control of amplification of the extracted DNA. The Real-time PCR assays were performed using the Real Time PCR Cycler Rotor Gene 3000 (Qiagen, Hilden Germany). Real Time PCR was considered positive for *Leishmania* DNA when an amplification curve at threshold cycle (tC) below 40 was present.

### Statistical analysis

Descriptive statistics were used to analyze demographic and clinical characteristics. Chi-squared test was used to analyze potential correlation between detection of *Leishmania* infection and gender. Significance was established at p < 0.05.

## Results

We obtained clinical samples from 119 ESRD adult patients in dialysis treatment at the St.Orsola-Malpighi University Hospital (Bologna, Italy). Seventy-one out of 119 patients (60%) were potential candidates to kidney transplant. The age of patients ranged from 20 to 94 years, with a mean age of 70, with a male-to-female ratio of 73:46. Most of patients were Caucasian (92%), with a percentage of 2% of Asian, 5% of African and 1% of unknown origin. The main causes of ESRD in the cohort of patients were diabetic nephropathy (28%) and hypertensive vascular nephropathy (18%). Twenty-three percent of patients had a nephropathy of unknown origin. Demographic and clinical data of the study cohort are reported in Table 1.

**Table 1:** Demographic variables and clinical data of patients with end stage renal disease

	Total (119 pts^a^)	No^b^ of positive pts to* Leishmania* infection (19)	No. of negative pts to* Leishmania* infection (100)
Age, years (range)			
20–34	2 (2%)	1 (5%)	1 (1%)
35–49	8 (7%)	0 (0%)	8 (8%)
50–64	23 (19%)	5 (26%)	18 (18%)
65–79	51 (43%)	7 (37%)	44 (44%)
> 80	33 (28%)	6 (32%)	27 (27%)
Unknown	2 (2%)	0 (0%)	2 (2%)
Mean age, years	70	69	79
Gender			
Female	46 (39%)	5 (26%)	41 (41%)
Male	73 (61%)	14 (74%)	59 (59%)
Origin			
Caucasian	110 (92%)	17 (89%)	93 (93%)
Asian	2 (2%)	0 (0%)	2 (2%)
African	6 (5%)	2 (11%)	4 (4%)
Unknown	1 (1%)	0 (0%)	1 (1%)
Cause of ESRD			
Diabetic nephropathy	33(28%)	4 (21%)	29 (29%)
Glomerular disease	13 (11%)	2 (11%)	11 (11%)
Hypertensive vascular nephropathy	21 (18%)	6 (32%)	15 (15%)
Interstitial disease	16 (13%)	1 (5%)	15 (15%)
Polycystic kidney disease	9 (8%)	1 (5%)	8 (8%)
Unknown	27 (23%)	5 (26%)	22 (22%)
Dialysis vintage, years (range)			
0–3	47 (39%)	10 (53%)	37 (37%)
4–7	35 (29%)	4 (21%)	31 (31%)
7–10	17 (14%)	2 (11%)	15 (15%)
> 10	20 (17%)	3 (16%)	17 (17%)

Percentages are calculated based on the total number for each column

^a^Pts; patients, ^b^No.; number

Nineteen out of 119 patients (15.9%) were positive at the serological and/or molecular test for *Leishmania* infection; the mean age of positive patients was 69 years and the male-to-female ratio was 14:5. There was no significant correlation between presence of asymptomatic *Leishmania* infection and gender (χ2 = 1.45, *p* value = 0.23). None of the Asian patients was positive for the parasitic infection, while 33% of African patients and 15% of Caucasian patients were positive for at least one *Leishmania* test. Results of WB analysis showed the presence of specific antibody to 14 and/or 16 kDa proteins, indicating the presence of anti-*Leishmania* IgG in 17 ESRD patients (14.3-95% CI 8—20.4%) (Table 2). In 3 patients’ sera, both bands were detected, while antibodies against p14 or p16 were found in 13 and 1 serum sample, respectively. Circulating *Leishmania* kDNA was detected in peripheral blood from 3 out of 119 ESRD patients (2.5–95% CI 0.3–5.3%); only 1 patient showed simultaneous detection of anti-leishmanial IgG (WB reactivity to 14 kDa protein) and parasitic DNA in peripheral blood. None of the patients testing positive to anti-leishmanial antibody and/or parasitic DNA showed signs or symptoms compatible with VL or tegumentary leishmaniasis.

**Table 2:** Western Blot and Real Time PCR to detect asymptomatic *Leishmania* infection in patients with end stage renal disease (n = 119)

WB Pos	WB Neg	PCR Pos	PCR Neg
No. (%)	No. (%)	No. (%)	No. (%)
17 (14.3)	102 (85.7)	3 (2.5)	116 (97.5)

WB; Western Blot. PCR; polymerase chain reaction. Pos; positive. Neg; negative. No; number

## Discussion

To our knowledge, there are no studies on the prevalence of asymptomatic *Leishmania* infection in kidney transplant candidates. Here, we detected the presence of clinically silent *Leishmania* infection in 15.9% of ESRD patients in dialysis treatment. As 60% of these patients were potential recipients for kidney transplant, this study group might be considered as a model for candidates to SOT.

*Leishmania*-specific antibodies were revealed by WB in serum samples obtained from 17 patients, which showed antibody to 14 and/or 16 kDa proteins, while parasitic DNA was detected in blood samples of 3 patients by kDNA amplification. Overall, our results suggest an important cumulative exposure to *Leishmania* in the examined cohort, which may potentially lead to parasitic reactivation upon transplantation.

A recent study from southern Spain showed a prevalence of 4.8% of asymptomatic *Leishmania* infection in renal transplant recipients [13]; differently from this study, we enrolled dialyzed patients who did not receive immunosuppressive drugs. The lower prevalence of *Leishmania* infection observed in the Spanish study as compared to our study can be related to a variable circulation of the parasite in different endemic areas or to diversity of methods employed for parasite screening. Considering the latter possibility, the indirect immunofluorescence assay (IFAT) employed by Elmahallawy and colleagues could have underestimated the infection rate in asymptomatic carriers. In fact, evidence indicates that IFAT exhibits a lower sensitivity than WB and sera from VL patients recognize the 14–16-kD bands in WB at concentrations 10^7^ lower than the threshold values of IFAT [11]. As using a combination of tests has been reported to increase the capacity to detect asymptomatic *Leishmania* infection [1], we employed the most sensitive serological method, namely WB [9, 11, 14] together with a molecular test.

*Leishmania* infection may be transmitted to transplant recipients by different ways, including the donor allograft, the transfusion of blood products, a de novo posttransplant infection or through reactivation of pretransplant infection, the latter generally considered the most common route of infection [2, 15]. Nevertheless, studies focusing on parasite route of transmission in SOT are lacking. In addition, there is no consensus recommendation to screen the organ transplant recipients for *Leishmani*a infection in endemic areas [2, 5, 9]. Furthermore, different methods are available to detect asymptomatic *Leishmania* infection, including serology, molecular tests and ex vivo whole blood stimulation with parasitic antigens combined with specific cytokine release assay [1, 16], but no gold standard method has been defined so far [5, 17]. On the other hand, updated guidelines recommend that those known to be *Leishmania*-seropositive at the time of transplant should be monitored closely for potential reactivation of the parasitic infection [5].

Considering the high prevalence of *Leishmania* infection among potential kidney transplant recipients detected in the current study, we emphasize the importance of further evaluating sensitive tools for screening this hemoflagellate protozoan in SOT candidates in endemic areas.
